# Factors Associated with Ruptured Ectopic Pregnancy: A 10-Year Review at a District Hospital in Ghana

**DOI:** 10.1155/2022/1491419

**Published:** 2022-03-07

**Authors:** Promise E. Sefogah, Nana E. Oduro, Alim Swarray-Deen, Hanson G. Nuamah, Raphael B. Takyi, Mercy A. Nuamah, Samuel A. Oppong

**Affiliations:** ^1^Department of Obstetrics & Gynaecology, University of Ghana Medical School, Accra, Ghana; ^2^Department of Obstetrics & Gynaecology, Korle Bu Teaching Hospital, Accra, Ghana; ^3^Department of Epidemiology and Disease Control School of Public Health, University of Ghana, Accra, Ghana; ^4^Department of Obstetrics & Gynaecology, LEKMA Hospital, Teshie, Accra, Ghana

## Abstract

**Background:**

Approximately 1–2% of all pregnancies are ectopic. Despite a decline in ectopic pregnancy-related mortality, there is still a paucity of information on the factors associated with clinical presentation and outcomes in Sub-Saharan Africa which is essential in determining the most appropriate treatment modalities.

**Methods:**

We performed a ten-year retrospective chart review of cases of ectopic pregnancies managed at the Lekma hospital and assessed them for peculiar risk factors, clinical presentation, and outcomes. Associations between patients' sociodemographic characteristics, clinical presentation, and treatment outcome were evaluated using multiple logistic regression and reported as adjusted odds ratios (AOR). The confidence interval (CI) was set at 95%, and a *p* value <0.05 were considered significant.

**Results:**

Over the ten-year period, there were 115 ectopic pregnancies and 14,450 deliveries (7.9/1,000). The mean age ± standard deviation of the 115 patients was 27.61 ± 5.56. More than half of the patients were single (59/115, 51.3%). The majority (71.3%) of the patients presented with a ruptured ectopic pregnancy. After adjusting for covariates, the odds of an ectopic pregnancy presenting as ruptured among single patients was 2.63 times higher than that of married patients (AOR = 3.63, 95% CI: 1.33–9.93, *p*=0.01). Ectopic pregnancies located in the isthmic region of the tube had a 77% lower odds of presenting as ruptured than those located in the ampullary region (AOR = 0.23, 95% CI: 0.07–0.74, *p*=0.01). The odds of rupturing were 1.69 times increased for every additional week after the missed period (AOR = 2.69, 95% CI: 1.56–4.64, *p* < 0.01). No mortalities were reported as a result of an ectopic pregnancy.

**Conclusion:**

Most of the cases of ectopic pregnancy presented ruptured. Marital status and period of amenorrhoea were significantly associated with rupture.

## 1. Introduction 

Ectopic pregnancy, defined as the implantation of the embryo outside the uterine cavity, is a common, life-threatening gynaecological emergency. Worldwide, approximately 1–2% of all naturally conceived pregnancies result in ectopic implantation [1,2]. Studies in Africa also reported a similar incidence (1–2%) of ectopic pregnancies [3–5]. A three-year (2013–2015) retrospective review of all gynaecological admissions in a referral hospital in the Volta Region of Ghana reported an ectopic pregnancy prevalence of 2.1% [6].

Broadly, ectopic pregnancies may be tubal, nontubal, or heterotopic pregnancy, with tubal being the most common [7, 8]. In nontubal pregnancies, oocytes are fertilized at sites other than the fallopian tube, or the oocytes can be fertilized within the fallopian tube but extruded into the peritoneal cavity [9, 10]. Heterotopic pregnancies refer to the coexistence of an intrauterine pregnancy with an ectopic pregnancy. They are extremely rare and are primarily associated with assisted reproductive technology treatments [11, 12].

Although all sexually active women of childbearing age are at risk of ectopic pregnancy, different studies have identified several risk factors for its occurrence. Studies have established a link between ectopic pregnancy and maternal factors such as age [13, 14], previous ectopic pregnancy [13, 15, 16], and history of pelvic infections [1, 17, 18]. In addition, ectopic pregnancies have been associated with infertility and infertility treatment [13, 14, 19], including assisted reproductive techniques [20–22] and tubal reconstructive surgery [19, 23, 24]. Other risk factors are smoking [23, 25] and contraceptive use [13].

Treatment options for ectopic pregnancy include medical, laparoscopic, and laparotomy. Although the treatment success rate of ectopic pregnancy varies among studies, several studies have established no significant differences between the treatment methods [26–29].

Despite significant declines in ectopic pregnancy-related mortality, a four-year (2004–2008) autopsy review found that ectopic pregnancy accounted for 8.7% of maternal mortality and ranked among the top 5 causes of maternal death in Ghana [30]. In addition, ectopic pregnancy may also give rise to several psychoemotional problems [31, 32] and financially burden both patients and healthcare institutions [33]. Currently, there is still a paucity of information on the factors associated with clinical presentation and outcomes in our subregion, which is essential in determining the most appropriate treatment modalities. Therefore, the objective was to conduct an epidemiological analysis of cases of ectopic pregnancy managed at the Lekma hospital to determine associated factors, patterns in presentation, treatment modalities, and outcomes.

This study differs from other ectopic pregnancy-related studies conducted in Ghana as it analyzed a broader epidemiological aspect of archived clinical data to determine associations between various sociodemographic characteristics of the population seen, clinical presentation, and treatment outcomes.

## 2. Methods

This ten-year retrospective cohort study analyzed archived data of ectopic pregnancies at the Ledzokuku-Krowor Municipal Assembly (LEKMA) Hospital, Ghana. LEKMA is a 100-bed capacity district hospital that conducts approximately 2,000 deliveries annually. Regularly, there have been at least 3 specialists, 2 medical officers, and 5 house officers at the Obstetrics and Gynaecology Department. The study population consisted of all ectopic pregnancy cases managed at the hospital between 2010 and 2019. Outcome variables were the type of ectopic pregnancy, presentation (tubal, nontubal, and heterotopic), treatment received, period of amenorrhea (counting from their missed period), and clinical outcomes (ruptured or unruptured). The independent variables were sociodemographic characteristics, including age, education level, smoking, assisted reproductive techniques, contraception, medical history of patients, and clinical findings.

The data were collected from the archived medical, laboratory, and surgical notes with a structured questionnaire. This was thoroughly inspected for completeness before the information was transferred into Microsoft Excel 2018 (Microsoft Company, USA) and exported into STATA (version 16.1) for analysis. Associations between treatment outcome and patients' demographic characteristics and clinical findings were analyzed using multiple logistic regression and reported as crude odds ratios (OR) and adjusted odds ratios (AOR). The confidence interval (CI) was set at 95%, and *p* values <0.05 were considered significant. Approval to conduct this study was obtained from the Ethical and Protocol Review Committee of the College of Health Sciences, University of Ghana (protocol identification number: CHS–Et/M3-5.15, 2019–2020).

## 3. Results

Over the ten-year period, there were 115 ectopic pregnancies and 14,450 deliveries reported at the Lekma Hospital, giving rise to approximately 0.08 ectopic pregnancies per delivery (7.9/1,000); all ectopic pregnancies were included in the study. The mean age of the patients was 27.6 years (±5.56), with the majority (52.2%) of them being 25–34 years. Over forty per cent of the study group were nulliparous, and 51.3% were not married. The mean period of amenorrhoea at diagnosis was 10.3 weeks (±1.12). Over ninety per cent of the patients had no previous abdominal surgery (Table 1). All the ectopic pregnancies seen at this facility were managed surgically via salpingectomy.

There were 82 cases (71.3%) of ruptured ectopic pregnancies documented in the review. There was a significantly larger proportion of single women experiencing ruptured ectopic compared to married women (47/59 (80%) vs. 35/56 (62.5%)). The majority (17/18, 94.4%) of the patients experienced ectopic rupture after ten weeks of amenorrhea. Most (40/64, 62.5%) of the ectopic pregnancies occurred in the ampulla, and these had the highest proportion (40/51, 78.4%) of rupture. There was a single case of ruptured infundibulum ectopic pregnancy (Table 2).

After adjusting for covariates, the odds of an ectopic pregnancy presenting as ruptured among single patients was 2.63 times higher than that of married patients (AOR = 3.63, 95% CI: 1.33–9.93, *p*=0.01). Ectopic pregnancies located at the isthmic region of the tube had a 77% lower odds of presenting as ruptured than those located at the ampullary region (AOR = 0.23, 95% CI: 0.07–0.74, *p*=0.01). The odds of rupturing increased 1.69 times for every additional week after their missed period (AOR = 2.69, 95% CI: 1.56–4.64, *p* < 0.01) (Table 3). No mortalities were reported as a result of an ectopic pregnancy.

## 4. Discussion

This study reviewed ten years of archived data on ectopic pregnancies managed at the Lekma Hospital in Accra, Ghana, to determine patterns in presentation, treatment modalities, and outcomes, and to assess risk factors associated with ectopic pregnancy.

Our analysis demonstrated that the average period of amenorrhea was higher in patients presenting with ruptured ectopic pregnancies than in those with unruptured ectopic pregnancies. This finding is similar to other studies that reported higher gestational ages to be significantly associated with ruptured ectopic gestations. A retrospective study in Turkey found the odds of tubal rupture at gestational ages of 6–8 and greater than eight weeks to be 3.67 and 46.69, respectively [34]. Other researchers observed a positive association of borderline significance between rupture and gestational age [35]. This finding could be explained by the fact that the ectopic gestational mass generally increases with increasing gestational age, and rupture becomes more likely when the size of the gestational mass outgrows the fallopian tube. The exception is interstitial ectopic pregnancy which can expand until 15 weeks of gestation. Because of the problems associated with misdiagnosis of interstitial ectopic, they also have a higher rate of presenting with a ruptured tube with severe haemoperitoneum [35]. In our ten-year review, there was no case of interstitial ectopic gestation recorded.

In our study, marital status was significantly associated with ectopic rupture, with single women having three times higher odds of suffering a ruptured ectopic pregnancy than married women. We speculate that married couples are more likely to report earlier when they experience pregnancy symptoms and thus allow an earlier diagnosis of ectopic pregnancy when it is unruptured, which is then managed medically or surgically. Asah-Opoku et al., in an earlier study, demonstrated a higher ectopic pregnancy risk amongst single women [36]. Possible explanations were that, unlike married women who generally have a more stable intimate relationship, single women are more likely to have multiple sexual partners with the attendant risk of developing sexually transmitted diseases (STDs). STDs, including pelvic inflammatory disease, have been shown to be associated with ectopic pregnancy [37].

Parity has been shown to play a role in ruptured ectopic. Sindos et al. found a positive association between parity and ruptured ectopic pregnancy following their retrospective analysis of 19 years of archived data on ectopic pregnancies in Athens, Greece [35]. In contrast, we found no relationship between parity and ruptured ectopic gestation after adjusting for confounders in our study. Nonetheless, Sindos et al. found no significant association between ruptured ectopic and a patient's age, which agrees with our findings [35].

Even though the site of ectopic was found to be associated with a ruptured ectopic in the crude analysis, this was not found to be statistically significant in the adjusted model. Moreover, the mode of presentation, i.e., whether the patient presented with bleeding per vaginum or lower abdominal pain or both, or with sudden collapse, did not have any association with the outcome of a ruptured ectopic gestation. An earlier case-control study in Cameroon by Mindjah et al. has suggested that it is necessary to facilitate access to well-equipped healthcare facilities to enable the early diagnosis of ectopic pregnancies [38]. However, findings from this study have indicated strongly that despite the availability of this district facility, the majority of ectopic pregnancies present as ruptured. This may suggest some deficiencies in public health awareness and a lack of health-seeking behavior. Specifically, early reporting of secondary amenorrhea provides a crucial opportunity to diagnose ectopic pregnancies before they rupture and cause potentially life-threatening morbidity. This agrees with Goyaux et al., who conducted a 20-year systematic review of ectopic pregnancies in Africa and recommended that ectopic pregnancy should be considered a critical public health indicator in developing countries [39].

A significant limitation of this study was the use of archived routine data that did not capture some otherwise essential risk factors or potential confounders. However, the study's main strength was that the review covered ten years in a low-income setting and showed patterns that could be reviewed for future clinical and research uptake.

## 5. Conclusion

The majority of patients with ectopic pregnancies presented following a rupture. Marital status and period of amenorrhoea are significantly associated with ruptured ectopic gestation. This suggests a deficiency in public health awareness and health-seeking behaviour for early reporting of missed periods in our setting.

## Figures and Tables

**Table 1:** Demographic characteristics of study participants.

Characteristics	Frequency	Percentage
Age		
≤24	39	33.9
25–34	60	52.2
≥35	16	13.9

Parity (at the time of ectopic)		
0	50	43.5
1	33	28.7
≥2	32	27.8

Marital status		
Single	59	51.3
Married	56	48.7

Period of amenorrhea (weeks)		
≤8	36	31.3
9–10	61	53
≥11	18	15.7

Previous abdominal surgery		
Yes	8	7
No	107	93

Mean age = 27.61 (standard deviation = 5.56).

**Table 2:** Relationship between participant's characteristics and ectopic rupturing.

Characteristics	Ectopic outcome		*P* value
	Ruptured	Unruptured	
	*n* (%)	*n* (%)	
Age			0.51
<25	30 (76.9)	9 (23.1)	
25–30	42 (70.0)	18 (30.0)	
>30	10 (62.5)	6 (37.5)	

Parity (at the time of ectopic)			0.10
0	39 (78.0)	11 (22.0)	
1	25 (75.8)	8 (24.2)	
≥2	18 (56.3)	14 (43.7)	

Marital status			0.03
Single	47 (79.7)	12 (20.3)	
Married	35 (62.5)	21 (37.5)	

Period of amenorrhea (weeks)			<0.01
≤8	17 (47.2)	19 (52.8)	
9–10	48 (78.7)	13 (21.3)	
≥11	17 (94.4)	1 (5.6)	

Previous abdominal surgery			0.69
Yes	5 (62.5)	3 (37.5)	
No	77 (72.0)	30 (28.0)	

Side affected			0.41
Left	34 (66.7)	17 (33.3)	
Right	48 (75.0)	16 (25.0)	

Site of ectopic			0.01
Tubal			
Ampullary	40 (78.4)	11 (21.6)	
Fimbrial	13 (59.1)	9 (40.9)	
Infundibulum	1 (100.0)	0 (0)	
Isthmic	11 (50.0)	11 (50.0)	

Nontubal			
Cornual	16 (94.1)	1 (5.9)	
Ovarian	1 (50.0)	1 (50.0)	

Mode of presentation			0.70
BPV	22 (66.7)	11 (33.3)	
LAP	12 (70.6)	5 (29.4)	
BPV/LAP	44 (72.1)	17 (27.9)	
Sudden collapse	4 (100.0)	0 (0)	

*n*: number in group; BPV: bleeding per vaginum; LAP: lower abdominal pain.

**Table 3:** Characteristics associated with ectopic outcome (rupturing).

Characteristics	Ectopic outcome (rupturing)			
	COR (95% CI)	*P* value	AOR (95% CI)	*P* value
Age	0.93 (0.87–1.00)	0.06	—	
Parity	0.70 (0.48–1.02)	0.06	—	

Marital status				
Married	Reference		Reference	
Single	2.35 (1.02–5.41)	0.04	3.63 (1.33–9.93)	0.01
Period of amenorrhea (weeks)	2.66 (1.62–4.37)	<0.01	2.69 (1.56–4.64)	<0.01

Previous abdominal surgery				
No	Reference		—	
Yes	0.65 (0.15–2.89)	0.57	—	

Side affected				
Left	Reference		—	
Right	1.50 (0.67–3.38)	0.33	—	

Site of ectopic				
Ampullar	Reference		Reference	
Isthmic	0.28 (0.09–0.80)	0.02	0.23 (0.07–0.74)	0.01
Cornual	4.4 (0.52–36.94)	0.17	—	
Fimbrial	0.40 (0.13–1.17)	0.09	—	
Ovarian	0.28 (0.02–4.76)	0.38	—	
Infundibulum	—	—	—	

Mode of presentation				
BPV	Reference		—	
LAP	1.20 (0.34–4.27)	0.78	—	
BPV and LAP	1.29 (0.52–3.23)	0.58	—	
Sudden collapse	—	—	—	

CI: confidence interval; COR: crude odds ratio; AOR: adjusted odds ratio; BPV: bleeding per vaginum; LAP: lower abdominal pain.

## Data Availability

The data used to support the findings of this study are available from the corresponding author upon request.
